# Prosocial Interventions and Health Outcomes: A Systematic Review and Meta-Analysis

**DOI:** 10.1001/jamanetworkopen.2023.46789

**Published:** 2023-12-08

**Authors:** Margaret Byrne, Rayner Kay Jin Tan, Dan Wu, Gifty Marley, Takhona Grace Hlatshwako, Yusha Tao, Jennifer Bissram, Sophie Nachman, Weiming Tang, Rohit Ramaswamy, Joseph D. Tucker

**Affiliations:** 1 Gillings School of Global Public Health, University of North Carolina at Chapel Hill; 2 University of North Carolina Project–China, Guangzhou, Guangdong, China; 3 Saw Swee Hock School of Public Health, National University of Singapore and National University Health System, Singapore, Singapore; 4 Clinical Research Department, London School of Hygiene and Tropical Medicine, London, United Kingdom; 5 Nuffield Department of Population Health, University of Oxford, Oxford, United Kingdom; 6 Health Sciences Library, University of North Carolina at Chapel Hill; 7 Department of Medicine, University of North Carolina at Chapel Hill; 8 Cincinnati Children’s Hospital Medical Center, Cincinnati, Ohio

## Abstract

**Importance:**

Prosocial interventions encourage voluntary actions that benefit others. Community solidarity in response to the COVID-19 pandemic, expanding mutual aid programs, and health workforce issues have accelerated prosocial health interventions.

**Objective:**

To investigate the association of prosocial interventions with health outcomes in clinical trials and observational studies.

**Data Sources:**

In this systematic review and meta-analysis informed by the *Cochrane Handbook for Systematic Reviews of Interventions*, 5 databases (MEDLINE [via PubMed], Embase, CINAHL, PsycInfo, and Scopus) were searched from database inception through February 23, 2023. The search included terms for altruism and prosocial behaviors, health outcomes, and study type.

**Study Selection:**

Included studies, determined by multiple reviewers, compared health outcomes in a prosocial intervention group with a nonintervention group.

**Data Extraction and Synthesis:**

Following the Preferred Reporting Items for Systematic Reviews and Meta-analyses guideline, data extraction and synthesis captured quantitative and qualitative data. To pool data from quantitative studies, random-effects meta-analyses were used to estimate the impact of prosocial interventions. To combine data from quantitative and qualitive studies, data were transformed into qualitative narratives using meta-aggregation.

**Main Outcomes and Measures:**

The main outcome was whether prosocial interventions were associated with improved health outcomes. Barriers to and facilitators of implementation of these interventions were assessed.

**Results:**

The search identified 5229 citations; 30 studies were included in the synthesis. Studies indicated that prosocial interventions were associated with positive health outcomes for givers (17 studies [56.7]) and recipients (8 [26.7%]). Prosocial interventions included acts of kindness (12 studies [40.0%]), cash gifts (7 [23.3%]), pay-it-forward approaches (6 [20.0%]), and expressions of kindness (5 [16.7%]). Improvements were reported in depression, testing for sexually transmitted diseases, vaccine uptake, physical activity, and individual biomarkers. Data from 6 studies (20.0%) demonstrated that pay-it-forward approaches were associated with increased uptake of diagnostic tests or vaccines among vulnerable groups (moderate certainty of evidence). Data from 14 studies (46.7%) suggested that community connectedness facilitated prosocial interventions. Shared vulnerabilities among groups (eg, sexual minority individuals, older adults) may provide a context for collective mobilization to improve health in local communities.

**Conclusions and Relevance:**

This systematic review and meta-analysis found that prosocial interventions were associated with improved health outcomes among vulnerable groups and have been useful for addressing health disparities. Further research is needed to develop and evaluate prosocial interventions.

## Key Points

**Question:**

Are prosocial interventions associated with improved health outcomes?

**Findings:**

This systematic review and meta-analysis of 30 studies found that prosocial interventions were associated with improved health outcomes among vulnerable groups and have been useful for addressing health disparities. Pay-it-forward approaches were associated with increased uptake of diagnostic tests or vaccines among vulnerable groups, and community connectedness facilitated prosocial interventions.

**Meaning:**

Prosocial interventions may generate benefits for both givers and recipients.

## Teaser

This systematic review and meta-analysis investigates associations between prosocial interventions and health outcomes as well as barriers to and facilitators of such interventions.

## Introduction

The tendency for humans to help each other is deeply rooted, providing an opportunity to organize prosocial interventions.^1^ Prosocial interventions encourage voluntary actions that benefit others.^2,3^ Prior to the COVID-19 pandemic, volunteerism was increasing in the US^4^ and evidence supporting prosocial interventions expanded.^5^ The COVID-19 pandemic nurtured mutual aid groups that often leverage prosocial tendencies. In addition, cynicism and burnout have become common in many clinical settings, increasing the rationale for prosocial interventions.^6^ Data from global surveys on generosity suggest that kindness increased during COVID-19–related restrictions.^7^ COVID-19 responses spurred the development of mutual aid programs, and related community-driven initiatives provided pathways for people to help each other locally^8^; many of these programs persisted after the pandemic.^9^

**Figure 1.**
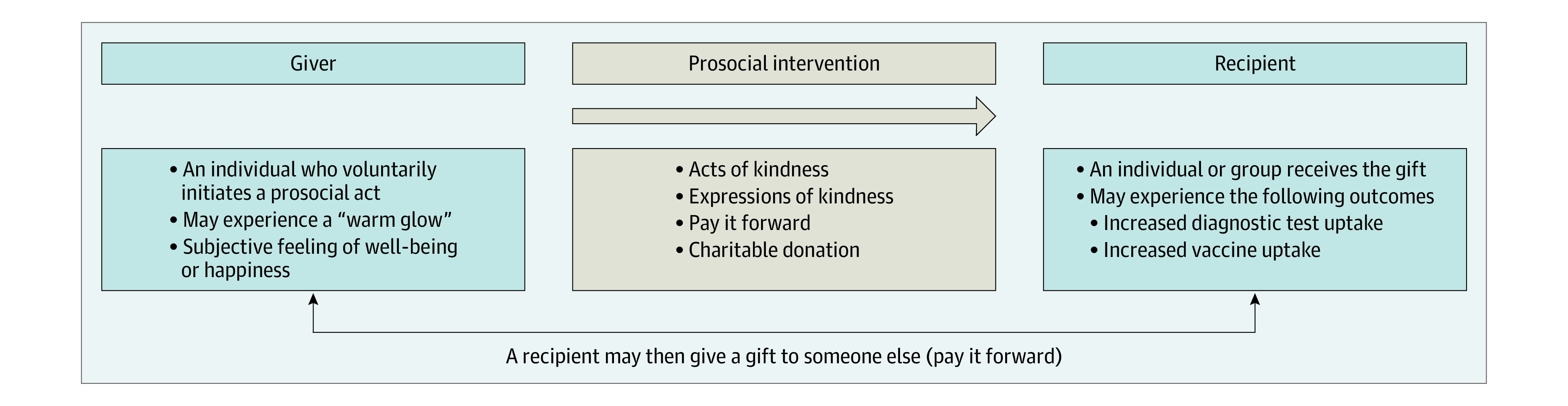
Overview of Prosocial Interventions From the Perspective of the Giver and Recipient

The number of clinical trials assessing the potential effect of prosocial interventions on health or medical outcomes is growing.^10,11^ A prosocial intervention is an intervention that encourages voluntary actions that benefit others (Figure 1). A prosocial intervention needs to have someone organizing it, but ultimately, it is voluntary or up to the individual whether to follow through. Prosocial interventions have been shown to improve mental^12^ and physical^13^ health. Such interventions include acts of kindness (actions that benefit someone else), pay-it-forward (receiving a gift and then giving a gift to someone else in the community^14^), and expressions of kindness (messages that benefit someone else). A meta-analysis demonstrated that volunteering among older adults was associated with 24% decreased mortality after adjustment for potential confounders.^15^ Another study found that among socially anxious college students, performing acts of kindness was associated with a decrease in social avoidance.^16^ During the COVID-19 pandemic, prosocial public health messages were associated with a higher level of adherence to self-isolation compared with other types of messages,^17^ and a study found that greater prosocial attitudes were associated with well-being (measured using the Mental Health Continuum–Short Form) across regions.^18^

The World Health Organization, a *Lancet* commission, and others have emphasized the need for strengthening prosociality within society.^19^ Despite the growing number of studies examining prosocial interventions, there have been few reviews.^5,20^ This systematic review and meta-analysis was conducted to synthesize worldwide evidence on the outcomes of prosocial interventions in health based on studies with comparator arms using mixed-methods approaches to better understand barriers to and facilitators of prosocial interventions. We were particularly interested in pay-it-forward approaches. These data may inform the development of prosocial interventions in health and be used to improve existing interventions.

## Methods

We used the Preferred Reporting Items for Systematic Reviews and Meta-Analyses (PRISMA) checklist to report the findings, and we organized the review according to the *Cochrane Handbook for Systematic Reviews of Interventions*.^21^ The review was registered in PROSPERO.

### Inclusion Criteria

To qualify for inclusion in this systematic review and meta-analysis, a study needed to have a prosocial intervention group and a comparator group. We only included studies with a comparator group because there are many single-arm descriptive studies that provide limited information about the efficacy of the intervention. Studies needed to be in the English language and published in a peer-reviewed journal. We only included studies reporting on health outcomes, defined as outcomes relating to mental health and well-being, public health and disease prevention, and physical health. Included studies were randomized or nonrandomized studies with a comparator that assessed the association of a prosocial intervention with health outcomes.

### Search Strategy

We searched for studies and reviews in MEDLINE (via PubMed), Embase, CINAHL, PsycInfo, and Scopus published from database inception through October 29, 2021, and the search was updated on February 23, 2023. A medical librarian (J.B.) developed the search strategy (eAppendix in Supplement 1). The search included terms for altruism and prosocial behaviors (eg, *pay-it-forward*, *acts of kindness*, *generosity*), health outcomes (eg, *public health*, *health behavior*, health messag*), and study type (developed from a validated search filter for controlled trials).^22^ We used medical subject headings or Emtree subject headings when appropriate and subject terms when applicable. An additional search for ongoing clinical trials was conducted in ClinicalTrials.gov.

### Study Selection

The title and abstract of each study were each reviewed by 1 reviewer (M.B., R.T., D.W., G.M., or T.G.H.). Then a full text review was conducted by 2 independent reviewers (M.B., R.T.). When the reviewers agreed, the decision was finalized. If the 2 reviewers did not agree on a study, a third independent reviewer (J.D.T.) made the final decision. We used the Cochrane Collaboration’s tool to assess risk of bias in randomized clinical trials.^23^ For nonrandomized studies, the Risk of Bias in Nonrandomized Studies of Interventions tool was used to assess the risk of bias.^24^ Qualitative and quantitative data from the selected studies were extracted manually by 3 reviewers (M.B., R.T., and T.G.H.). The evidence extracted in this review was qualitative in nature, obtained by extracting quantitative information and converting it into qualitative statements and by extracting qualitative findings. Three reviewers (M.B., R.T., and T.G.H.) extracted data for the 30 studies. Data for all studies were extracted in duplicate, and therefore, each of the 3 reviewers extracted data for 20 studies.

### Statistical Analysis

#### Meta-Analysis

We used random-effects meta-analysis to generate pooled relative risks and risk differences with 95% CIs for the studies that had within-study comparisons of pay-it-forward vs non–pay-it-forward approaches. We also conducted a meta-analysis of the pooled impact of prosocial interventions using weighted mean differences for studies that adopted standardized outcomes of depressive symptoms, anxiety, positive affect, negative affect, and psychological well-being. The *I*^2^ statistic was used to measure statistical heterogeneity between studies. Analyses were performed in Stata, version 16.1 (StataCorp LLC).

We used the Grading of Recommendations Assessment, Development, and Evaluation (GRADE) approach to assess the confidence of findings in our random-effects meta-analysis.^25^ While there was substantial heterogeneity in health outcomes, we proceeded to use the GRADE approach based on the recommendations in the Cochrane handbook. If there were comparable outcomes and interventions, we pooled the data and conducted a meta-analysis. We had to have at least 3 studies to pool the interventions. Our assessments were based on our pooled outcomes specifically on the association of pay-it-forward interventions with the uptake of health behaviors. We then assessed the risk of bias, imprecision, inconsistency, indirectness, and any potential issues to examine the certainty of such evidence associated with these outcomes of interest.

#### Mixed-Methods Synthesis

We used a mixed-methods synthesis because it allowed us to combine themes from all qualitative and quantitative data and consider the findings together. Our data synthesis consisted of 3 parts: a transformation of quantitative and mixed-methods results to qualitative narratives, a databased convergent synthesis, and a meta-aggregation interpretation.^26^ We completed this synthesis by first describing the main findings as a qualitative narrative to facilitate comparison between studies. First, we completed a table of the central findings from each quantitative study. We also extracted the central qualitative findings from qualitative and mixed-methods studies. In addition to the main findings, we extracted secondary findings from quantitative data into qualitative form. The central finding was taken directly from the text as the main finding from the study. The secondary findings were statements made by the authors that did not directly answer the research question but were still included because they were relevant to the research question in this review. The reviewers identified central findings first by looking at the concluding statement in the abstract and discussion section of the article. Then 1 individual reviewer repeated multiple reads of the studies and identified secondary finding statements to include from the results and discussion sections.

We used a mixed-methods synthesis for analysis.^27^ There was substantial heterogeneity in the research designs and outcomes, and this method allowed us to synthesize the literature into distinct findings. The qualitative findings were analyzed using meta-aggregation techniques. The basis of meta-aggregation is that the researchers do not attempt to reinterpret the included studies but rather categorize and present the findings of the studies.^26^ We determined that categorized findings would be the most useful outputs of this review because these categories consolidate the information from the review into facilitators of or barriers to implementation of prosocial interventions. This was to further understand how prosocial interventions can influence health outcomes. The reviewers grouped the qualitative statements (representing both qualitative and quantitative findings) to form categories. The categories provide information on the overarching focus of the group of findings. We then further aggregated the categories to develop broader themes.

Informed by the JBI SUMARI approach, the reviewers generated statements that were an aggregation of the themes created.^28^ This final step resulted in domains that incorporate evidence from 2 or more themes to describe factors that impact the effectiveness of prosocial interventions.

## Results

**Figure 2.**
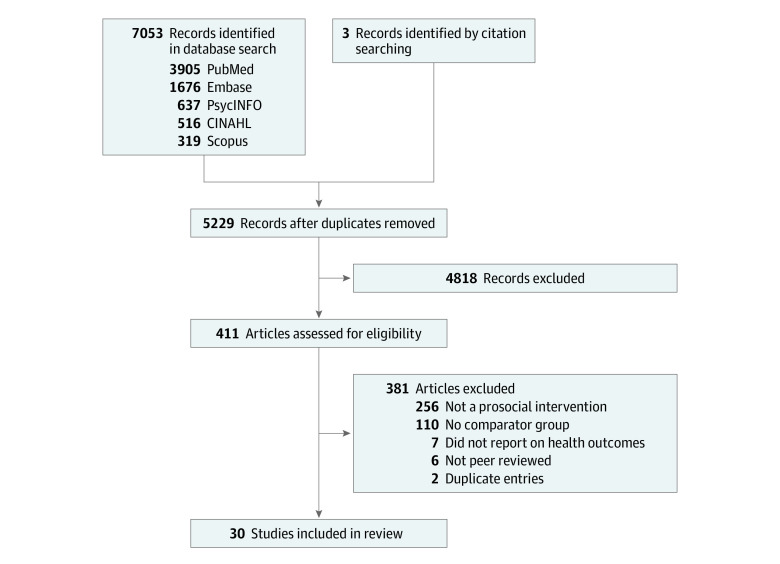
PRISMA Diagram PRISMA indicates Preferred Reporting Items for Systematic Reviews and Meta-Analyses.

The initial search yielded 7053 citations. After removing duplicates, there were 5229 citations. After title and abstract screening, 411 full-text studies were assessed for eligibility. The majority of these studies (n = 381) were excluded, most (n = 366) because they were not prosocial interventions or lacked a comparator group. Ultimately, 30 studies were included in the literature review (Figure 2 and eTable 1 in Supplement 1).

We identified 24 randomized clinical trials,^12,29,30,31,32,33,34,35,36,37,38,39,40,41,42,43,44,45,46,47,48,49,50,51^ 3 nonrandomized studies,^52,53,54^ and 3 mixed-methods studies.^55,56,57^ Most of the studies (17 [56.7%]) analyzed mental health and well-being as the primary health outcome.^12,29,30,31,34,36,38,41,45,47,48,49,50,51,53,56,57^ Other studies examined disease screening (5 studies [16.7%]^43,44,46,52,55^), physical activity (2 [6.7%]^32,33^), and biomarkers (alanine transaminase, conserved transcriptional response to adversity [*CTRA*], leukocyte telomere length) (3 [10.0%]^31,37,42^) (Table 1). Nineteen studies (63.3%) were in high-income countries,^29,30,31,32,33,34,36,37,38,39,40,41,42,45,49,51,53,56,57^ and none were in low-income countries. Eight studies (26.7%) were in middle-income countries.^43,44,46,47,50,52,54,55^ Three studies (10.0%) were conducted in multiple countries.^12,35,48^ Four studies (13.3%) focused on prosocial interventions during the COVID-19 pandemic.^12,47,50,54^

**Table 1.** Summary of Study Characteristics

Characteristic	Studies, No. (%) (N = 30)
Type of prosocial intervention	
Acts of kindness	12 (40.0)
Expressions of kindness	5 (16.7)
Pay it forward	6 (20.0)
Charitable donations for participation	3 (10.0)
Prosocial spending on others	4 (13.3)
Recipients of prosocial intervention	
Individuals	17 (56.7)
Groups or populations	13 (43.3)
Health outcomes of prosocial interventions	
Mental health and well-being	17 (56.7)
Disease screening	5 (16.7)
General public health	3 (10.0)
Physical activity	2 (6.7)
Individual biomarkers	3 (10.0)
Countries studied	
US	9 (30.0)
China	8 (26.7)
Canada	4 (13.3)
The Netherlands	4 (13.3)
Japan	1 (3.3)
Hong Kong	1 (3.3)
Colombia and Chile	1 (3.3)
US and Canada	1 (3.3)
Multinational	1 (3.3)
Study design	
Randomized clinical trial	24 (80.0)
Nonrandomized	3 (10.0)
Mixed methods	3 (10.0)

We identified 5 types of prosocial interventions—acts of kindness, charitable donation, prosocial spending, expressions of kindness, and pay-it-forward. Twelve studies (40.0%) focused on acts of kindness.^12,29,31,34,35,36,37,39,40,41,45,57^ A total of 7 studies included cash gifts to others,^32,33,42,47,48,49,50^ with 3 (10.0%) focused on charitable donations^32,33,42^ and 4 (13.3%) on prosocial spending.^47,48,49,50^ Five studies (16.7%) focused on expressions of kindness,^30,38,51,53,56^ and 6 (20.0%) used a pay-it-forward approach.^43,44,46,52,54,55^

Of the 12 studies with a focus on acts of kindness,^12,29,31,34,35,36,37,39,40,41,45,57^ 8 (66.7%) found that acts of kindness were associated with improved health outcomes^29,35,36,37,39,40,41,45^ and 4 (33.3%) found no significant differences.^12,31,34,57^ Acts of kindness were associated with improved mental health and well-being and biomarker (*CTRA* expression) outcomes.^37^ No studies reported that acts of kindness were associated with worse health outcomes.

Incorporating prosocial spending (7 studies [23.3%]) in prosocial interventions to improve health outcomes had mixed results.^32,33,42,47,48,49,50^ Six studies (20.0%) found an improvement in health outcomes associated with prosocial spending,^32,42,47,48,49,50^ and 1 study (3.3%) found short-term improvements that did not last.^33^ Prosocial spending interventions were positively associated with mental health and well-being, physical activity, and biomarker (alanine transaminase) outcomes (indicating an improvement in nonalcoholic fatty liver disease).^32,43,47,48,49,50^

There was mixed evidence on whether expressions of kindness were effective in improving health outcomes (5 studies [16.7%]^30,38,51,53,56^). Two studies examined the health outcomes of expressions of kindness for well-being and found a positive association.^58,59^

Six studies (20.0%) evaluated pay-it-forward interventions.^43,44,46,52,54,55^ In these studies, individuals received a gift and were asked if they would like to give a gift to someone else in their community. Five pay-it-forward studies (83.3%) focused on sexually transmitted disease (STD) test uptake,^43,44,46,52,55^ while 1 (16.7%) measured influenza vaccination.^54^ Among the studies focusing on STD test uptake, the population of interest was either men who have sex with men (4 studies [80.0%]^43,46,52,55^) or female sex workers (1 study [20.0%]^44^). For pay-it-forward studies, the evidence showed that a pay-it-forward approach was associated with an increase in test uptake for gonorrhea and chlamydia in men who have sex with men and in female sex workers as well as vaccine uptake for influenza among children and older adults in China. ^43,44,46,52,54,55^ The results of the risk of bias assessments can be found in eTables 2 and 3 in Supplement 1.

### Results of Meta-Analysis

**Figure 3.**
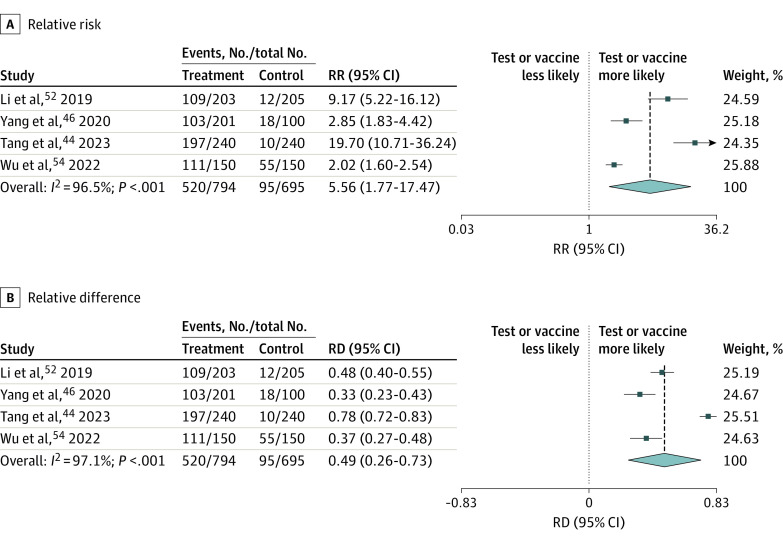
Meta-Analysis of Within-Study Comparisons of Pay-It-Forward vs Non–Pay-It-Forward Arms for the Relative Risk (RR) and Relative Difference (RD) of Receiving a Diagnostic Test or Vaccine The group using the pay-it-forward approach was the treatment arm, and the group that did not use this approach was the control arm. Weights are from random-effects analysis.

Four studies (13.3%) compared a pay-it-forward intervention group with another group (Figure 3),^44,46,52,54^ and these data were pooled and meta-analyzed. The pay-it-forward approach was associated with a significant increase in the likelihood of receiving a diagnostic test or vaccine compared with standard of care (pooled risk ratio, 5.56 [95% CI, 1.77-17.47]); risk difference, 0.49 [95% CI, 0.26-0.73]) (Figure 3). Our GRADE approach for the random-effects meta-analysis is summarized in eTable 4 in Supplement 1. We found a moderate level of certainty in studies reporting that pay-it-forward interventions led to an uptake of diagnostic tests or vaccines among vulnerable groups.

Two studies (6.7%) compared using kindness interventions with not using kindness interventions and measured the same mental health outcomes: depressive symptoms and psychological well-being.^34,45^ Additionally, 3 studies (10.0%) compared using vs not using kindness interventions and measured anxiety symptoms and positive and negative affect.^29,41,57^ Data from meta-analyses suggest that the weighted mean differences were not statistically significant (eFigure in Supplement 1).

### Results of Mixed-Methods Evidence Synthesis

Overall, our review identified 25 studies (83.3%) that showed prosocial interventions were associated with improvements in health outcomes^29,32,33,35,36,37,38,39,40,41,42,43,44,45,46,47,48,49,50,51,52,53,54,55,56^ and 5 studies (16.7%) in which they were not associated with a significant improvement in health outcomes.^12,30,31,34,57^ A total of 22 (73.3%) studies examined the association of such interventions with health outcomes for the givers^12,29,30,31,32,33,34,35,36,37,38,40,41,42,45,48,49,50,51,53,56,57^; 17 studies (56.7%) found an association with positive health outcomes for givers^29,32,33,35,36,37,38,40,41,42,45,48,49,50,51,53,56^ and 5 (16.7%) found no association.^12,30,31,34,57^ In contrast, a total of 8 studies (26.7%) also examined the association of prosocial interventions with health outcomes for the recipients, all of which found a positive association.^39,43,44,46,47,52,54,55^ Among the 17 studies (56.7%) that reported on mental health or well-being outcomes,^12,29,30,31,34,36,38,41,45,47,48,49,50,51,53,56,57^ 12 (70.6%) reported a positive association.^29,36,38,41,45,47,48,49,50,51,53,56^ Among the 13 studies (43.3%) that focused on other outcomes, all 13 reported a positive association.^32,33,35,37,39,40,42,43,44,46,52,54,55^

Prosocial interventions were also shown to have the potential to positively impact community solidarity among participants. Community solidarity is the individual feeling of belonging to a specific group. A total of 7 studies (23.3%) found that participating in a prosocial intervention led to a strengthening of one’s relationship with another individual or a community.^29,34,41,43,44,52,53^ For example, in a pay-it-forward model, participants indicated that in addition to the free gonorrhea or chlamydia test they were receiving, they felt cared for by others in their community.^52^

The results from the meta-analysis suggest that not all prosocial intervention approaches were equally effective in improving health outcomes. Using the data from this review, we identified some potential mechanisms that could increase effectiveness in prosocial interventions. We used a meta-aggregation process to identify these mechanisms. We identified 12 categories, which were then synthesized into 5 themes: community and connection, external influences affecting health outcomes, individual interest, internal drive, and emotional expression (Table 2). These 5 themes were then aggregated into 2 domains: individual factors and interpersonal or contextual factors that affect the implementation of prosocial interventions.

**Table 2.** Themes Derived From Meta-Aggregation

Domain, theme, category	Findings
**Interpersonal and contextual factors impacting the effect of prosocial interventions**	
Community and connection	
Community	The model substantially increased test uptake compared with the standard of care. From a financial perspective, most of the costs associated with testing were supported by local MSM, suggesting a viable pathway to sustainable service delivery.^52^ The Kind Acts procedure significantly increased participants’ satisfaction with their social relationships and reduced their concern with negative social outcomes compared with the activity monitoring control and, to a lesser extent, performing behavioral experiments.^29^ Pay-it-forward spurred community engagement by actively engaging MSM in the development and implementation of the service.^52^ The findings indicate that cognitive behavioral techniques taught in a peer group with additional parent training and a focus on prosocial intentions and responsibility of children are effective for children with psychosocial problems.^53^ Participants described feeling a sense of belonging and experiencing fewer feelings of loneliness.^41^ Engaging in other-focused kindness may enhance perceptions of social support among breast cancer survivors.^34^ Community solidarity among MSM in China can be characterized by 3 factors: engagement, social network support, and sense of belonging.^43^ Positive affect can be increased in individuals with high levels of social anxiety, and positive affect enhancement strategies like performing kind acts may result in wider social benefits.^29^
Connection to recipient	The major finding of this study was that in the group that received an appreciative letter and questionnaire and, later, a brochure about the bone marrow registry, the percentage of blood donors who joined the bone marrow registry was 2.0 times greater than in the control group of blood donors who received only the brochure and 2.2 times greater than in the control group of blood donors who received neither the questionnaire nor the brochure.^39^ Positive reactions of people toward the participants were likely to strengthen the effects of the acts of kindness.^36^ Many participants preferred performing acts of kindness for friends and family because they were able to see the immediate impact in comparison with performing the acts for strangers.^57^ Prosocial behavior for strong social ties could boost mental well-being more than performing kind acts for weak social ties or unspecified social ties.^45^ The results of this study suggest that the donor’s perceived relationship to a blood center may play an influential role in how he or she responds to a request it might make.^39^ Participants stated that they experienced gratification, improved mood, and increased happiness on seeing the positive reactions of recipients of deliberate acts of kindness.^41^ Gratitude was associated with higher self-esteem and lower depression among left-behind children in China.^47^ Sense of belonging was higher in the pay-it-forward intervention arm and may be associated with the uptake of a gonorrhea or chlamydia test.^43^ In this study, many intervention-group participants noted that seeing the reaction of the acts-of-kindness recipient improved their well-being (ie, increased their happiness, improved their overall mood, made them feel good, and encouraged them to continue engaging in the behavior).^57^
External influences affecting health outcomes	
Negative affect	An increase of psychological distress was detected in the whole sample throughout the intervention independent of the kindness condition.^45^
Atmosphere and context	The critical component to enhance the efficacy of the program was the creation of a prosocial and empathetic atmosphere in classrooms. Based on the proposed model, program effectiveness would be affected by inner contextual factors such as the classroom and school characteristics.^56^ Moreover, this study revealed the importance of contextual factors, such as school policy and community culture, for prosocial behavior development.^56^ Participants in both groups may have experienced improvements in positive body image as a result of experiencing greater positive affect overall.^30^ Charity donation may be less self-threatening in a collective society where reciprocating charity donations is a social routine.^47^ The nature of kind acts and their intended recipient play a key role in shaping the genomic impact of kindness.^37^ Psychological benefits were larger when generous acts were unrelated to COVID-19.^50^ Prosocial acts, particularly when enacted for a cause not directly related to the pandemic, could be a fruitful avenue for improving well-being during a pandemic.^12^ The pay-it-forward strategy revealed substantial generosity and promoted responsibility among the female sex workers to change their testing behaviors.^44^ The specific context of receiving a generous gift is likely to facilitate implementation and build trust in the service.^46^
Improvement in all groups	The study revealed no statistically significant differences between groups. Rather, participants in both groups reported that their involvement in the study provided an overall positive experience.^57^ Participants in both groups experienced improvements in state functionality appreciation and state body appreciation, with effect sizes ranging from medium to large.^30^ The high rates of test uptake in the pay-what-you-want arm suggest that free testing itself might be responsible for a substantial portion of the test uptake effect.^46^
**Individual factors impacting the effect of prosocial interventions**	
Individual interest	
No individual improvement	Expressive helping did not benefit survivors in the cluster with low survivorship problems. There was probably little room for improvement among these survivors, who demonstrated normal functioning to mild impairment.^38^ None of the interventions led to changes in well-being or depressive symptoms (primary outcomes) compared with the daily activity-writing control.^34^ Those assigned to perform prosocial acts did not differ significantly in depression, anxiety, happiness, or the belief that their life had meaning and was valuable compared with those assigned to report their daily activities.^12^ Women who promoted positive body image to a friend did not experience a more positive body image compared with women in an active control group.^30^
Personal financial decisions	This randomized clinical trial showed that incentives that used donations to a charity of choice, personal financial incentives, or combined charity donations and personal financial incentives each increased older adults’ initial uptake of increased levels of walking. However, the effects decreased and were no longer significant after the interventions were discontinued.^33^ There were no significant differences between the monetary and the donation incentives conditions.^32^ When given the choice between personal financial incentives and charitable donations, as in the combined group, participants were more likely to keep their earnings than to donate or share them.^33^ Free testing may have driven the increased test uptake rates because of the zero-price effect.^44^ The absence of a difference between the 3 intervention groups (donations to a charity of choice, personal financial incentives, or combined charity donations and personal financial incentives) suggests that these interventions make be equally effective, though this study was powered only to show a difference between each intervention and the control group.^33^
Internal drive	
Internal benefits to giver	One of the possible reasons for the improvement in self-control may be direct physical contact with the service animals. Petting an animal can induce relaxing physiological and neurological responses, such as reduction in systolic blood pressure, lower heart rate, and reduction of cortisol, resulting in stabilizing emotions and a calming effect.^56^ Among those in the volunteer group, higher postintervention empathic concern and altruistic behaviors were associated with lower levels of cardiovascular risk markers (adjusting for baseline values). Those in the control group showed no such associations.^40^ Spending money on others promotes happiness more than spending money on oneself.^49^ Individuals who engaged in acts of kindness experienced a significant improvement in resilience, possibly attributable to the positive empathy experienced by participants.^41^ As hypothesized, intervention group participants experienced increased resilience and reduced social anxiety and negative effect and described improvements in mood. Specifically, there was a significant difference in resilience within the intervention group.^41^ Prosocial acts may provide small, lasting benefits to emotional well-being and mental health.^12^ Prosocial engagement—doing something kind for others rather than oneself—reduces *CTRA* gene expression.^37^ Prosocial (vs non-prosocial or proself) behavior led to higher levels of self-reported positive affect, empathy, and social connectedness.^50^ The pay-it-forward strategy has the potential to enhance chlamydia and gonorrhea testing for Chinese female sex workers.^44^ The preliminary results of this pilot study indicate positive effects of the animal-assisted, school-based humane education program.^56^
Internal motivators or factors	This study found that altruism also became the major intrinsic motivating factor in the dietary treatment of NAFLD.^42^ Promoting patients’ intrinsic motivation by incorporating a “donations for decreased alanine aminotransferase” prosocial behavior incentive into conventional dietary and exercise intervention may provide a means to improve NAFLD.^42^ The findings suggest that the reward experienced from helping others may be deeply ingrained in human nature, emerging in diverse cultural and economic contexts.^48^ The salubrious effects of prosocial behavior in the short term are not likely due to the inhibition of cellular aging (at least as indexed by telomere length).^31^
Internal reward	The findings provide early support for small monetary incentives and charitable donations for promoting physical activity in community settings.^32^ Exploratory analyses revealed a pattern of results suggesting that engagement in either kindness activity led to reductions in loneliness across time.^31^ Engaging in kind acts resulted in significant increases in positive affect that were sustained over the 4-wk period.^29^ Adolescents who volunteered to help others also benefited themselves, suggesting a novel way to improve health.^40^ *CTRA* gene regulation was most sensitive to the social target of kindness (other people vs oneself) rather than the production of kind acts per se, with favorable effects observed in those who performed kind acts for others but not in those who performed kind acts for themselves.^37^ Intervention group participants described an increase in self-esteem via engaging in acts of kindness. Many noted that they felt like a good person and/or better about themselves for having engaged in acts of kindness.^57^
Emotional expression	
Expressive helping	The central finding of this study was that expressive helping has positive effects on distress in part through participants’ higher expression of positive emotions, consistent with theory and research.^51^ Writing focused on peer helping was only beneficial when it was preceded by expressive writing; the combination appeared to be critical.^38^ Findings supported hypothesized benefits of expressive helping for physical symptoms and general distress among survivors with moderate to severe survivorship problems.^38^
Emotions	Positive psychological interventions seemed to foster positive emotions and academic engagement but did not decrease negative emotions.^36^ The kindness intervention had a positive influence on both positive emotions and academic engagement, though not long term.^36^ The results showed no effects on negative emotions in either of the 2 interventions.^36^ Positive emotions and self-esteem were not significant mediating variables in the study.^45^

Abbreviations: *CTRA*, conserved transcriptional response to adversity; MSM, men who have sex with men; NAFLD, nonalcoholic fatty liver disease.

### Individual Factors Associated With Prosocial Interventions

A total of 7 studies (23.3%) examined whether individual factors moderated the association of prosocial interventions with outcomes.^30,32,33,38,44,50,55^ Six studies (20.0%) found that vulnerable groups (eg, sexual minority individuals, children, and older adults) were willing and able to do prosocial activities.^43,44,46,52,54,55^ One pay-it-forward study found that participants with low income were just as willing to donate to others as participants with a higher income.^55^ One study noted that illness in givers negatively affected their capacity to help others, underscoring the importance of context.^38^

Three studies (10.0%) demonstrated the role that self-interest had in decisions to engage in prosocial interventions.^32,33,44^ Two studies (6.7%) noted that participants who received monetary incentives for themselves for a specific behavior experienced the same improvement in health outcomes as those whose action resulted in a donation to a charity of their choice.^32,33^

### Interpersonal and Contextual Factors and the Effects of Prosocial Interventions

The relationship between giver and recipient may impact the implementation of prosocial interventions. Our review identified several interpersonal and contextual factors that impacted the effectiveness of prosocial interventions. A total of 14 studies (46.7%) found that interpersonal and contextual factors facilitated prosocial interventions.^29,36,39,41,43,44,45,46,50,52,53,54,55,57^ These factors included the giver’s relationship to the recipient, the recipient’s response to the prosocial intervention, a broader connection to a community, and the community and policy environment.

Three studies (10.0%) found that a prior relationship between the giver and the recipient impacted the effectiveness of a prosocial intervention.^39,45,57^ Three studies (10.0%) found that positive recipient responses to a prosocial intervention improved the mental health of the giver.^36,41,57^ One study (3.3%) found that givers preferred to be generous with friends or family members, thus allowing them to directly see the impact.^57^ One study evaluating an act of kindness found that positive reactions among recipients enhanced health outcomes.^41^

In the 6 pay-it-forward studies, the interventions were developed using cocreation.^43,44,46,52,54,55^ Cocreation is the process of researchers working iteratively with end users to develop an intervention.^52^ Cocreation enhanced community participation in the development, implementation, and uptake of these interventions.^43,44,46,52,54,55^ Qualitative findings from a mixed-methods study on pay-it-forward gonorrhea and chlamydia testing among men who have sex with men also found that the intervention enhanced community identity among sexual minority individuals and increased their desire to help the community.^55^ The pay-it-forward intervention gave participants a platform to create a sense of belonging and a chance to help others in their local community.

Finally, 7 studies (23.3%) noted that the context in which the prosocial intervention took place had an impact.^12,30,43,46,47,50,56^ For example, a study focusing on prosocial interventions in children found that school policy and community culture were important factors in the outcomes of the intervention.^56^ Another study found that peer charity was associated with improved self-esteem and reduced depression among left-behind children in China (those who live in rural areas, far from one or both parents).^47^

## Discussion

Prosocial interventions can generate benefits for both the giver and the recipient. Our pooled data suggest that pay-it-forward approaches were associated with increased uptake of diagnostic tests and vaccines among men who have sex with men and female sex workers in China. Prosocial interventions may provide an opportunity to strengthen within-group ties and shared characteristics among marginalized groups. This study extends the literature by focusing on prosocial interventions, capturing data on health outcomes for the giver and recipient, and synthesizing data from quantitative and qualitative studies.

Most studies found that prosocial interventions were associated with positive health outcomes for givers and recipients. This finding aligns with other evidence on prosocial interventions and health outcomes.^10,11^ We believe that prosocial interventions can affect health outcomes because of the connected nature of humans and their inherent willingness to help each other.^60^ The COVID-19 pandemic may have encouraged the development of prosocial interventions due to the increased focus on social cohesion and altruistic behavior,^60^ but only 4 of our included studies were conducted during COVID-19–related restrictions.^12,47,50,54^

Our data suggest that community connectedness facilitated prosocial interventions. Previous research found that a connection to the recipient can influence prosocial behavior.^61^ Connection can result from shared interests and also shared vulnerabilities.^62^ One study explicitly used community connectedness as a mechanism to increase participation in prosocial interventions.^63^ The relationship between an individual and a larger community may play a role in motivating prosocial intervention. For example, sexual minority individuals who realize the disparities in health outcomes may be more likely to engage in a prosocial intervention. These findings align with the pooled findings indicating that the pay-it-forward approach was associated with increased STD testing. In these interventions, community engagement was a central component.

Our data suggest that pay-it-forward approaches were associated with increased test and vaccine uptake among vulnerable populations in China. This is consistent with the literature on financial and social incentives to enhance uptake of preventive services.^64,65^ Given that diagnostic tests and vaccines often require small fees, a pay-it-forward approach that makes these services free may be particularly effective among people with low income for whom a fee could limit access. Our data indicate that prosocial interventions may be particularly effective among vulnerable populations, which supports the findings from our pay-it-forward pooled results. Further research on transitioning pay-it-forward approaches from single ad hoc programs to enduring public health benefits is needed.

Our data have implications for research and policy. From a research perspective, pragmatic clinical trials are needed to examine prosocial interventions within existing health systems. Considerations for enhancing the outcomes of prosocial interventions can be found in eTable 5 in Supplement 1. This is important to understand how prosocial interventions could complement and extend health services for specific groups. Future research focusing on why some interventions work while others do not will be a useful contribution to the understanding of prosocial interventions. From a policy perspective, the free services provided as part of pay-it-forward interventions could inform incremental steps toward universal health coverage. Pay-it-forward also provides an innovative financing mechanism to galvanize support for health within local communities, decreasing reliance on external donors. Most of the studies in this review were conducted in high-income countries. We need further investigation into the use of prosocial interventions in low-income countries, specifically focusing on low-cost interventions.

### Limitations

Our review has several limitations. First, we excluded studies without a comparator. There may be important single-arm studies that have demonstrated the feasibility and acceptability of prosocial interventions. However, these study designs are less robust and limit the researchers’ ability to discern whether any outcomes resulted from the intervention. Second, all the pay-it-forward studies were conducted in China, underlining the need for pay-it-forward research outside China. Third, additional studies in resource-constrained settings are needed to understand how this approach could work in different contexts. Fourth, we did not focus on organ donation or related acts because the field of organ donation is moving toward an opt-out approach that makes individual decision-making less important.

## Conclusion

This systematic review and meta-analysis found that prosocial interventions have been associated with improved health outcomes among vulnerable groups and that these approaches have been useful for addressing health disparities. The innate tendency for humans to cooperate and help one another provides a strong basis for prosocial health interventions. Small acts of kindness can be contagious, rippling through communities and improving health along the way. Prosocial interventions may help improve health, generate funding support for health programs, and enhance collective responses to diseases. More pilot testing of prosocial interventions is required to better understand how to scale up these approaches.

## Notes

Supplement 1. **eAppendix.** Search Strategy for CINAHL, SCOPUS, PsychINFO, and Embase **eTable 1.** Summary of Studies **eTable 2.** Cochrane Collaboration’s Tool to Assess Risk of Bias in RCT Studies **eTable 3.** ROBINS-I Tool Results for Nonrandomized Studies **eTable 4.** GRADE Assessment and Certainty of Evidence for Random Effects Meta-Analysis **eTable 5.** Considerations for Enhancing the Effect of Prosocial Interventions **eFigure.** Meta-Analysis of Strong Kindness vs Nonkindness for the Weighted Mean Difference for the Effect on Depressive Symptoms, Anxiety Symptoms, Positive Affect, Negative Affect, and Psychological Well-Being

Supplement 2.
Data Sharing Statement
