# Supplementary material for: Prosocial Interventions and Health Outcomes: A Systematic Review and Meta-Analysis
Source: JAMA Netw Open. 2023 Dec 8;6(12):e2346789. doi: 10.1001/jamanetworkopen.2023.46789 (PMC10709779; doi:10.1001/jamanetworkopen.2023.46789)
Supplement: Supplement 1. — eAppendix. Search Strategy for CINAHL, SCOPUS, PsychINFO, and Embase eTable 1. Summary of Studies eTable 2. Cochrane Collaboration’s Tool to Assess Risk of Bias in RCT Studies eTable 3. ROBINS-I Tool Results for Nonrandomized Studies eTable 4. GRADE Assessment and Certainty of Evidence for Random Effects Meta-Analysis eTable 5. Considerations for Enhancing the Effect of Prosocial Interventions eFigure. Meta-Analysis of Strong Kindness vs Nonkindness for the Weighted Mean Difference for the Effect on Depressive Symptoms, Anxiety Symptoms, Positive Affect, Negative Affect, and Psychological Well-Being [file jamanetwopen-e2346789-s001.pdf]

Supplementary Online Content

Byrne M, Tan R, Wu D, et al. Prosocial interventions and health outcomes: a systematic review and meta-analysis. *JAMA Netw Open*. 2023;6(12):e2346789. doi:10.1001/jamanetworkopen.2023.46789

eAppendix. Search Strategy for CINAHL, SCOPUS, PsychINFO, and Embase
eTable 1. Summary of Studies
eTable 2. Cochrane Collaboration's Tool to Assess Risk of Bias in RCT Studies
eTable 3. ROBINS-I Tool Results for Nonrandomized Studies
eTable 4. GRADE Assessment and Certainty of Evidence for Random Effects Meta-Analysis
eTable 5. Considerations for Enhancing the Effect of Prosocial Interventions
eFigure. Meta-Analysis of Strong Kindness vs Nonkindness for the Weighted Mean Difference for the Effect on Depressive Symptoms, Anxiety Symptoms, Positive Affect, Negative Affect, and Psychological Well-Being

This supplementary material has been provided by the authors to give readers additional information about their work.

© 2023 Byrne M et al. *JAMA Network Open*.

eAppendix: Search Strategy for CINAHL, SCOPUS, PsychINFO, and Embase

CINAHL:

**CINAHL**			
**Search #**	**Query**	**Results**	
#1: Prosocial Subject terms	MH("beneficence" OR "gift giving" OR "altruism" OR "positive psychology")	7,686	8520
#2: Prosocial Title/Abstract	TI(prosocial OR prosociality OR pro-social OR pro-sociality OR pay-it-forward OR paying-it-forward OR acts-of-kindness OR act-of-kindness OR generosity OR beneficence OR benevolence OR gift-giving OR helping-behavior OR helping-behaviour OR helping-behaviours OR helping-behaviors OR kindness OR positive-psych* OR positive-emot* OR altruism OR altruistic OR gratitude OR humanitarianism OR charitable-behavior) OR AB(prosocial OR prosociality OR pro-social OR pro-sociality OR pay-it-forward OR paying-it-forward OR acts-of-kindness OR act-of-kindness OR generosity OR beneficence OR benevolence OR gift-giving OR helping-behavior OR helping-behaviour OR helping-behaviours OR helping-behaviors OR kindness OR positive-psych* OR positive-emot* OR altruism OR altruistic OR gratitude OR humanitarianism OR charitable-behavior)	14,366	16304

© 2023 Byrne M et al. *JAMA Network Open*.

#3: public health Subject terms	MH("Health Promotion" OR "Public Health" OR "Health Behavior" OR "Mental Health")	205,663	228339
#4: public health Title/abstract	TI(public-health OR community-health OR health-behavior OR health-behaviors OR health-behaviour OR health-behaviours OR health-promotion OR disease-prevention OR health-messag* OR physical-health OR mental-health) OR AB(public-health OR community-health OR health-behavior OR health-behaviors OR health-behaviour OR health-behaviours OR health-promotion OR disease-prevention OR health-messag* OR physical-health OR mental-health)	277,148	313422
#5: Study type[1]	(MH randomized controlled trials OR MH double-blind studies OR MH single-blind studies OR MH random assignment OR MH pretest-posttest design OR MH cluster sample OR MH Quasi-Experimental Studies OR TI (randomised OR randomized) OR AB (random*) OR TI (trial) OR (MH (sample size) AND AB (assigned OR allocated OR control)) OR MH (placebos) OR PT (randomized controlled trial) OR AB (control W5 group) OR MH (crossover design) OR MH (comparative studies) OR AB (cluster W3 RCT)) NOT ((MH animals+ OR MH animal studies OR TI animal model*) NOT MH human)	869,879	962108
#6	(S1 OR S2) AND (S3 OR S4) AND S5	407	474

© 2023 Byrne M et al. *JAMA Network Open*.

[1] Source: Glanville J, Dooley G, Wisniewski S, Foxlee R, Noel-Storr A. Development of a search filter to identify reports of controlled clinical trials within CINAHL Plus. Health Information & Libraries Journal. 2019 Mar;36(1):73-90. Modified to include quasi-experimental studies.

© 2023 Byrne M et al. *JAMA Network Open*.

PSYCINFO

**PSYCINFO**			
**Search #**	**Query**	**Results**	
#1: Prosocial Subject terms	DE("Kindness" OR "Gratitude" OR "Charitable Behavior" OR "Altruism" OR "Positive Psychology" OR "Prosocial Behavior" OR "Positive Emotions")	25,051	28640
#2: Prosocial Title/Abstract	TI(prosocial OR prosociality OR pro-social OR pro-sociality OR pay-it-forward OR paying-it-forward OR acts-of-kindness OR act-of-kindness OR generosity OR beneficence OR benevolence OR gift-giving OR helping-behavior OR helping-behaviour OR helping-behaviours OR helping-behaviors OR kindness OR positive-psych* OR positive-emot* OR altruism OR altruistic OR gratitude OR humanitarianism OR charitable-behavior) OR AB(prosocial OR prosociality OR pro-social OR pro-sociality OR pay-it-forward OR paying-it-forward OR acts-of-kindness OR act-of-kindness OR generosity OR beneficence OR benevolence OR gift-giving OR helping-behavior OR helping-behaviour OR helping-behaviours OR helping-behaviors OR kindness OR positive-psych* OR positive-emot* OR altruism OR altruistic OR gratitude OR humanitarianism OR charitable-behavior)	48,885	53846
#3: public health Subject terms	DE(“Public Health” OR “Community Health” OR “Health Behavior” OR “Health Promotion” OR “Mental Health”)	172,054	189923

© 2023 Byrne M et al. *JAMA Network Open*.

#4: public health Title/abstract	TI(public-health OR community-health OR health-behavior OR health-behaviors OR health-behaviour OR health-behaviours OR health-promotion OR disease-prevention OR health-messag* OR physical-health OR mental-health) OR AB(public-health OR community-health OR health-behavior OR health-behaviors OR health-behaviour OR health-behaviours OR health-promotion OR disease-prevention OR health-messag* OR physical-health OR mental-health)	285,354	314810
#5: Study type	(DE "Randomized Controlled Trials" OR DE "Randomized Clinical Trials" OR DE "Quasi Experimental Methods" OR TI (randomised OR randomized) OR AB (random*) OR TI (trial) OR (MH (sample size) AND AB (assigned OR allocated OR control)) OR MH (placebos) OR PT (randomized controlled trial) OR AB (control W5 group) OR MH (crossover design) OR MH (comparative studies) OR AB (cluster W3 RCT)) NOT ((MH animals+ OR MH animal studies OR TI animal model*) NOT MH human)	305,310	326334
#6	(S1 OR S2) AND (S3 OR S4) AND S5	524	637

© 2023 Byrne M et al. *JAMA Network Open*.

SCOPUS

SCOPUS			
**Search #**	**Query**	**Results**	
#1: prosocial Title/abstract	TITLE-ABS(prosocial OR prosociality OR pro-social OR pro-sociality OR pay-it-forward OR paying-it-forward OR acts-of-kindness OR act-of-kindness OR generosity OR beneficence OR benevolence OR gift-giving OR helping-behavior OR helping-behaviour OR helping-behaviours OR helping-behaviors OR kindness OR positive-psych* OR positive-emot* OR altruism OR altruistic OR gratitude OR humanitarianism OR charitable-behavior)	72,908	84811
#2: public health Title/abstract	TITLE-ABS(public-health OR community-health OR health-behavior OR health-behaviors OR health-behaviour OR health-behaviours OR health-promotion OR disease-prevention OR health-messag* OR physical-health OR mental-health)	720,539	832476
#3: study types Title/abstract	TITLE-ABS((randomised OR randomized) W/3 (trial OR trials)) OR TITLE-ABS((single OR double OR doubled OR triple OR tripled OR treble OR treble) W/3 (blind* OR mask*)) OR TITLE-ABS(quasi-experiment*)	636,080	706443
#4	#1 AND #2 AND #3	251	319

© 2023 Byrne M et al. *JAMA Network Open*.

PubMed

**PubMed**			
**Search #**	**Query**	**Results**	
#1: prosocial Subject terms	(beneficence[mesh] OR gift-giving[mesh] OR helping-behavior[mesh] OR altruism[mesh] OR positive-psychology[mesh])	14,388	15016
#2: prosocial Title/abstract	(prosocial[tiab] OR prosociality[tiab] OR pro-social[tiab] OR pro-sociality[tiab] OR pay-it-forward[tiab] OR paying-it-forward[tiab] OR acts-of-kindness[tiab] OR act-of-kindness[tiab] OR generosity[tiab] OR beneficence[tiab] OR benevolence[tiab] OR gift-giving[tiab] OR helping-behavior[tiab] OR helping-behaviour[tiab] OR helping-behaviours[tiab] OR helping-behaviors[tiab] OR kindness[tiab] OR positive-psych*[tiab] OR positive-emot*[tiab] OR altruism[tiab] OR altruistic[tiab] OR gratitude[tiab] OR humanitarianism[tiab] OR charitable-behavior[tiab])	27,678	32317
#3: public health Subject terms	(public-health[mesh] OR health-behavior[mesh] OR health-promotion[mesh] OR mental-health[mesh])	8,657,140	9258184
#4: public health Title/abstract	(public-health[tiab] OR community-health[tiab] OR health-behavior[tiab] OR health-behaviors[tiab] OR health-behaviour[tiab] OR health-behaviours[tiab] OR health-promotion[tiab] OR disease-prevention[tiab] OR health-messag*[tiab] OR physical-health[tiab] OR mental-health[tiab])	582,451	670134

© 2023 Byrne M et al. *JAMA Network Open*.

#5	((randomized controlled trial[pt]) OR (controlled clinical trial[pt]) OR (randomized[tiab] OR randomised[tiab]) OR (placebo[tiab]) OR (quasi-experimental[tiab]) OR (randomly[tiab]) OR (trial[tiab]) OR (groups[tiab])) NOT (animals[mh] NOT humans[mh])	2,904,199	3179856
#6	(#1 OR #2) AND (#3 OR #4) AND #5	3,449	3900

© 2023 Byrne M et al. *JAMA Network Open*.

EMBASE

EMBASE			
**Search #**	**Query**	**Results**	
#1: prosocial Subject terms	(prosocial-behavior/exp OR generosity/exp OR beneficence/exp OR benevolence/exp OR gift-giving/exp OR kindness/exp OR altruism/exp OR gratitude/exp OR humanitarianism/exp OR positive-psychology/exp)	13,414	15019
#2: prosocial Title/abstract	(prosocial-behavior:ab,ti OR prosocial:ab,ti OR prosociality:ab,ti OR pay-it-forward:ab,ti OR pro-social:ab,ti OR pro-sociality:ab,ti OR pay-it-forward:ab,ti OR paying-it-forward:ab,ti OR acts-of-kindness:ab,ti OR act-of-kindness:ab,ti OR generosity:ab,ti OR beneficence:ab,ti OR benevolence:ab,ti OR gift-giving:ab,ti OR helping-behavior:ab,ti OR helping-behaviour:ab,ti OR helping-behaviours:ab,ti OR helping-behaviors:ab,ti OR kindness:ab,ti OR positive-psych*:ab,ti OR altruism:ab,ti OR altruistic:ab,ti OR gratitude:ab,ti OR humanitarianism:ab,ti OR charitable-behavior:ab,ti OR positive-emot*:ab,ti)	31,609	35842
#3: public health Subject terms	(public-health/exp OR health-behavior/exp OR health-behaviors/exp OR health-promotion/exp OR mental-health/exp)	897,711	993467
#4: public health Title/abstract	(public-health:ab,ti OR community-health:ab,ti OR health-behavior:ab,ti OR health-behaviors:ab,ti OR health-behaviour:ab,ti OR health-behaviours:ab,ti OR health-promotion:ab,ti OR disease-prevention:ab,ti OR health-messag*:ab,ti OR physical-health:ab,ti OR mental-health:ab,ti)	642,695	735541

© 2023 Byrne M et al. *JAMA Network Open*.

#5: study types	'randomized controlled trial'/exp OR 'controlled clinical trial'/exp OR 'quasi experimental study'/exp OR randomized:ti,ab OR randomised:ti,ab OR placebo:ti,ab OR randomly:ti,ab OR trial:ti,ab OR groups:ti,ab	4,702,586	5141511
#6	(#1 OR #2) AND (#3 OR #4) AND #5	1,411	1676
#7	#6 NOT ('conference abstract'/it OR 'conference paper'/it OR 'conference review'/it OR 'editorial'/it OR 'letter'/it OR 'short survey'/it)	1,127	

© 2023 Byrne M et al. *JAMA Network Open*.

eTable 1. Summary of studies

S/N	Lead Author / Year	Title	Country	Prosocial Nature of Intervention	Study Design	Study Hypotheses	Health Outcome Assessed	Statistical Analysis Conducted	Key finding
1	Aknin 2013	Prosocial spending and well-being: cross-cultural evidence for a psychological universal	Multi-country	Prosocial spending	4 Studies: Correlational analysis and experimental studies	Humans around the world experience hedonic benefits from generous spending.	Wellbeing, happiness, positive affect	Various regression analysis	Reward experienced from helping others may be deeply ingrained in human nature, emerging in diverse cultural and economic contexts.
2	Alden 2013	If it makes you happy: Engaging in kind acts increases positive affect in socially anxious individuals	Canada	Acts of kindness	Randomized controlled trial	Engaging in acts of kindness toward others could produce sustained increases in positive affect (PA) in highly socially anxious individuals.	Positive affect	Regression analysis	Positive affect can be increased in individuals with high levels of social anxiety and PA enhancement strategies like performing kind acts may result in wider social benefits.

© 2023 Byrne M et al. *JAMA Network Open*.

3	Alleva 2021	I appreciate your body, because... Does promoting positive body image to a friend affect one's own positive body image?	The Netherlands	Expressions of kindness	Randomized controlled trial	Promoting positive body image to a friend could lead to improvements in one's own positive body image.	Personal body appreciation	Separate one-way analyses of covariance	Women who promoted positive body image to a friend did not experience a more positive body image compared to women in an active control group. Instead, participants in both groups experienced improvements in state functionality appreciation and state body appreciation, with effect sizes ranging from medium to large.
4	Dunn 2008	Spending money on others promotes happiness	United States	Prosocial spending	Experimental methodology	People who receive an economic windfall experience greater happiness after receiving the windfall if they spend it on others.	Happiness	Regression analysis predicting happiness	Spending money on others promotes happiness more than spending money on oneself

© 2023 Byrne M et al. *JAMA Network Open*.

5	Fritz 2021	Kindness and cellular aging: A pre-registered experiment testing the effects of prosocial behavior on telomere length and well-being	United States	Acts of kindness	Single-blind, three-arm randomized controlled trial	A 4-week kindness intervention could slow leukocyte telomere short-ening and increase well-being.	#1 Life satisfaction, #2 flourishing, #3 loneliness, #4 Leukocyte Telomere Length (LTL)	Linear mixed-effects model analysis	The salubrious effects of prosocial behavior in the short term are not likely due to the inhibition of cellular aging (at least as indexed by telomere length). However, extending kindness to others holds promise as a future research direction for interventions to alleviate loneliness.
6	Galarraga 2020	Small sustainable monetary donation-based incentives to promote physical activity: A randomized controlled trial	United States	Charitable donations resulting from participation	Three-arm randomized controlled trial	Delivering small monetary and donation-based incentives within a community setting can promote physical activity	Days of attendance at the YMCA	Quantile regression	The findings provide early support for small monetary incentives and charitable donations for promoting PA in community settings.
7	Harkins 2017	A trial of financial and social incentives to increase older adults' walking	United States	Charitable donations resulting from participation	Four-arm randomized controlled trial	Social goals and donations can improve older adults' initial uptake and short-term retention of increased levels of walking.	Mean proportion of days that step-per-day goals were achieved during the 16-week intervention period	Generalized estimating equations	Incentives that use donations to a charity of choice, personal financial incentives, or combined charity donations and personal financial incentives each increase older adults' initial uptake of increased levels of walking. However, the effects decreased and were no longer significant after the interventions were discontinued.

© 2023 Byrne M et al. *JAMA Network Open*.

8	Haydon 2022	Kindness interventions for early-stage breast cancer survivors: An online, pilot randomized controlled trial	United States	Acts of kindness	Randomized controlled trial	Participants in each of the three kindness conditions (acts of kindness to others, acts of kindness to self, and selfkindness meditation) would demonstrate beneficial effects, from pre- to post-intervention, relative to those in the control condition	Psychological well-being and depressive symptoms	ANOVA	Among breast cancer survivors, performing prosocial acts may enhance feelings of social support.
9	Hu 2022	Peer charity donation, gratitude, and self-esteem among left-behind children	China	Prosocial spending (peer charity)	School-based intervention	Charity donations from non-LBC (left behind children) classmates should increase gratitude among the LBC. Gratitude should be associated with higher self-esteem, less depression, and less social anxiety among the LBC	Gratitude, self-esteem, depression	Structural equation modeling analysis	Gratitude was associated with higher self-esteem and lower depression among LBC.

© 2023 Byrne M et al. *JAMA Network Open*.

10	Li 2019	Pay-it-forward strategy to enhance uptake of dual gonorrhea and chlamydia testing among men who have sex with men in China: a pragmatic, quasi-experimental study	China	Paying-it-forward	Quasi-experimental pragmatic study	Pay-it-forward interventions can improve gonorrhea and chlamydia testing among Chinese MSM.	Test uptake	Chi-squared test and logistic regression	The model substantially increased test uptake compared to the standard of care. From a financial perspective, most of the costs associated with testing were supported by local MSM, suggesting a viable pathway to sustainable service delivery
11	Li 2020	A secondary mixed methods analysiss of a pay-it-forward gonorrhea/chlamydia testing program among men who have sex with men in China	China	Paying-it-forward	Sequential explanatory mixed methods design	Paying-it-forward can drive test uptake and donations, and socioeconomic factors may correlate with such outcomes	Test uptake	Logistic regression and multinomial regression	This study leveraged the original RCT and quasi-experimental papers (Li 2019 and Yang 2020) to explore factors associated with test uptake among participants in the pay-it-forward arms, and found that this intervention can reduce cost barriers, leverage generosity and reciprocity, and mobilize community altruism.

© 2023 Byrne M et al. *JAMA Network Open*.

12	LuengoKanacri 2020	Cross-national evidences of a school-based universal programme for promoting prosocial behaviours in peer interactions: Main theoretical communalities and local unicity	Colombia and Chile	Acts of kindness	Randomized controlled trial	Prosocial behavior can lower physical aggression in a school-based universal intervention adapted in two different (non-Western) countries, Colombia and Chile.	Prosocial behaviorPhysical Aggression	Analysis of covariance (ANCOVA)	The improvement on prosocial behaviours in both countries predicted significantly lower level of physical aggression.
13	Miles 2022	Using prosocial behavior to safeguard mental health and foster emotional well-being during the COVID-19 pandemic: A registered report of a randomized trial	United States and Canada	Acts of kindness	3-week experimental interention	Prosocial behavior increases happiness and the sense of meaning in life. Prosocial beahvior reduces depression and axiety.	Happiness, sense of meaning in life, depression, anxiety	Random intercept model	Prosocial acts may provide small, lasting benefits to emotional well-being and mental health.
14	Ngai 2021	Effectiveness of a school-based programme of animal-assisted humane education in Hong Kong for the promotion of social and emotional learning: A quasi-experimental pilot study	Hong Kong	Expressions of kindness	Sequential mixed-methods formative evaluation	Animal-assisted, school-based humane education programme can promote a humane attitude and enhance social–emotional competence.	social and emotional competence	Paired sample t tests and a mixed ANOVA	The preliminary results of this pilot study indicate positive effects of the programme.

© 2023 Byrne M et al. *JAMA Network Open*.

15	Ouweneel 2014	On being grateful and kind: results of two randomized controlled trials on study-related emotions and academic engagement	The Netherlands	Acts of kindness	Randomized controlled trial	A positive psychological intervention can enhance study-related positive emotions and academic engagement, and reduce study-related negative emotions.	Positive and negative emotions	RM-ANOVA analysis	The kindness intervention had a positive influence on both positive emotions and academic engagement, though not in the long run. The results showed no effects on negative emotions in either of the two interventions.
16	Regan 2022	The genomic impact of kindness to self vs. others: A randomized controlled trial	United States	Acts of kindness	Randomized controlled trial	A 4- week kindness intervention can reduce expression of a stress-related immune response gene signature known as the Conserved Transcriptional Response to Adversity (CTRA).	A stress-related immune response gene signature known as the Conserved Transcriptional Response to Adversity (CTRA).	Mixed effect linear models	Prosocial engagement—doing something kind for others rather than oneself—reduces CTRA gene expression.
17	Rini 2014	Harnessing benefits of helping others: a randomized controlled trial testing expressive helping to address survivorship problems after hematopoietic stem cell transplant	United States	Expressions of kindness	4- arm randomized controlled trial	Expressive helping (EH) intervention can reap benefits around survivorship problems among cancer survivors.	general distress, self-reported physical symptoms, HRQOL	Analysis of covariance (ANCOVA)	Findings supported hypothesized benefits of expressive helping for physical symptoms and general distress among survivors with moderate to severe survivorship problems.

© 2023 Byrne M et al. *JAMA Network Open*.

18	Sarason 1993	Increasing participation of blood donors in a bone-marrow registry	United States	Blood donation	Randomized controlled trial	Recognizing blood donors' contributions and involving them in a blood-center-related activity can increase the rate of participation in the bonw-marrow registry.	Joining the bone marrow registry	Logistic regression	The percentage of blood donors who joined the bone-marrow registry was 2.0 times greater than the control group of blood donors who received only the brochure and 2. 2 times greater than the control group of blood donors who received neither the questionnaire nor the brochure
19	Schreier 2013	Effect of volunteering on risk factors for cardiovascular disease in adolescents: a randomized controlled trial	Canada	Volunteering	Randomized controlled trial	Volunteering with elementary school–aged chil- dren can improve adolescents' cardiovascular risk profiles.	Cardiovascular risk markers of C-reactive protein level, interleukin 6 level, total cholesterol level, and body mass index	Analysis of covariance	Adolescents who volunteer to help others also benefit themselves, suggesting a novel way to improve health.
20	Shillington 2021	Kindness as an Intervention for Student Social Interaction Anxiety, Affect, and Mood: The KISS of Kindness Study	Canada	Acts of kindness	Repeated, mixed-methods design	Performing acts of kindness can improve affect, social interaction anxiety, and mood of undergraduate students.	Affect, social interaction anxiety and mood of students	Quantitative: ANOVA Qualitative: Summative content analysis	The study revealed no statistically significant differences between groups. Rather, participants in both groups reported that their involvement in the study provided an overall positive experience.

© 2023 Byrne M et al. *JAMA Network Open*.

21	Shillington 2021 II	Kindness as an Intervention for Student Social Interaction Anxiety, Resilience, Affect, and Mood: The KISS of Kindness Study II	Canada	Acts of kindness	Randomized controlled trial	Deliberate acts of kindness (DAKs) can impact stress-related outcomes of resilience, social interaction anxiety, affect, and mood of undergraduate and graduate students.	Resilience, social interaction anxiety, affect	Quantitative: Pearson's chi-squared test and mixed-model ANOVA Qualitative: Inductive content analysis	Intervention group participants experienced increased resilience, reduced social anxiety and negative afect, and described improvements in mood. Specifcally, there was a signifcant diference in resilience within the intervention group.
22	Sumida 2014	The 'donations for decreased ALT (D4D)' prosocial behavior incentive scheme for NAFLD patients	Japan	Charitable donations resulting from participation	Randomized controlled trial	Prosocial behavior incentives can serve as an effective intrinsic motivational factor in comparison with conventional dietary and exercise intervention alone for non-alcoholic fatty liver disease (NAFLD) patients.	ALT decrease after 12 weeks	Mann–Whitney U-test or Wilcoxon’s ranked sum test	Promoting patients’ intrinsic motivation by incorporating ‘D4D’ prosocial behavior incentive into conventional dietary and exercise intervention may provide a means to improve NAFLD.

© 2023 Byrne M et al. *JAMA Network Open*.

23	Sung 2022	Development of a Psychometric Tool to Measure Community Solidarity Among Sexual Minorities: Evidence From a Pay-it-Forward Randomized Controlled Trial	China	Paying-it-forward	Randomized controlled trial	Community solidarity means would be higher in the pay-it-forward intervention group and correlate with higher uptake of STI testing.	STD testing uptake	Factor analysis	Community solidarity among MSM in China can be charac- terized by 3 factors: engagement, social network support, and sense of belonging.
24	Tang 2023	A Pay-It-Forward Approach to Improve Chlamydia and Gonorrhea Testing Uptake Among Female Sex Workers in China: Venue-Based Superiority Cluster Randomized Controlled Trial	China	Pay-it-forward	Randomized controlled trial	Pay-it-forward is effective at increasing access to chlamydia and gonorrhea testing among female sex workers in China.	Chlamydia and gonorrhea test uptake	Generalized estimating equations	The pay-it-forward strategy has the potential to enhance chlamydia and gonorrhea testing for Chinese female sex workers.
25	Varma 2022	Prosocial behavior promotes positive emotion during the COVID-19 pandemic	China	Prosocial spending	Experimental	Prosocial behavior leads to higher levels of self- reported positive affect, empathy and social connectedness.	Well-being	Analysis of Covariance (ANCOVA)	Prosocial (vs. non-prosocial or proself) behavior led to higher levels of self-reported positive affect, empathy and social connectedness.

© 2023 Byrne M et al. *JAMA Network Open*.

26	Vliek 2014	I want to behave prosocially and I can choose to do so: Effectiveness of TIGER (Kanjertraining) in 8- to 11-year-olds	The Netherlands	Expressions of kindness	Quasi-experimental design	TIGER reduces psychosocial problems in eight- to eleven-year-olds in a mental health-care setting.	psyhosocial problem behavior	MANOVAS	Results indicated that TIGER significantly reduced externalizing and internalizing problems.
27	Wieners 2021	To whom should i be kind? A randomized trial about kindness for strong and weak social ties on mental wellbeing and its specific mechanisms of change	The Netherlands	Acts of kindness	Three-arm randomized controlled trial	A 4-week acts-of-kindness intervention targeting different types of social ties differ in impact on students' mental wellbeing, positive relations, depressive symptoms, anxiety, and perceived stress.	Mental wellbeing, positive relations, depressive symptoms, anxiety, and perceived stress	2-tailed tests with a significance level < .05	Prosocial behaviour for strong social ties could boost mental wellbeing more than performing kind acts for weak social ties or unspecified social ties.
28	Williamson 2017	Helping Yourself by Offering Help: Mediators of Expressive Helping in Survivors of Hematopoietic Stem Cell Transplant	United States	Expressions of kindness	Experimental	Word use reflective of emotional expression, cognitive processing, and change in perspective mediates the effects of expressive helping.	Psychological distress and physicial symptom bother	Linguistic Inquiry and Word Count and multi-level modeling	The central finding of this study is that expressive helping carries its positive effects on distress in part through participants' higher expression of positive emotions, consistent with theory and research.

© 2023 Byrne M et al. *JAMA Network Open*.

29	Wu 2022	Effectiveness of a pay-it-forward intervention compared with user-paid vaccination to improve influenza vaccine uptake and community engagement among children and older adults in China: a quasi-experimental pragmatic trial	China	Paying-it-forward	Quasi-experimental design	Pay-it-forward interventions can improve influenza vaccine uptake among children and older adults in China and spur donations	Vaccine uptake	Multivariables regression models	The study found that the pay-it-forward approach improved influenza vaccine uptake among children and older adults. It also spurred greater confidence and perceived importance of such vaccines, and led to an overall lower economic cost per vaccine due to the donations from participants who paid it forward.
30	Yang 2020	Pay-it-forward gonorrhoea and chlamydia testing among men who have sex with men in China: a randomised controlled trial	China	Paying-it-forward	Randomized controlled trial	Pay-it-forward interventions can improve gonorrhea and chlamydia testing among Chinese MSM.	Test uptake	Descriptive analysis and generalised estimating equations	The pay-it-forward strategy can increase gonorrhoea and chlamydia testing uptake among Chinese MSM and could be a useful tool for scaling up preventive services that carry a mandatory fee.

© 2023 Byrne M et al. *JAMA Network Open*.

eTable 2: Cochrane Collaboration's tool to assess risk of bias in RCT studies

	Lead Author/ Year	Title	Selection Bias	Performance Bias	Detection Bias	Attrition Bias	Reporting Bias
1	Aknin 2013	Prosocial spending and well-being: cross-cultural evidence for a psychological universal	High	Low	Unclear	Low	Low
2	Alden 2013	If it makes you happy: Engaging in kind acts increases positive affect in socially anxious individuals	Unclear	Low	Low	Low	Low
3	Alleva 2021	I appreciate your body, because... Does promoting positive body image to a friend affect one's own positive body image?	Low	Low	Low	Low	Low
4	Dunn 2008	Spending money on others promotes happiness	Unclear	Low	Low	Unclear	Unclear
5	Fritz 2021	Kindness and cellular aging: A pre-registered experiment testing the effects of prosocial behavior on telomere length and well-being	Low	Low	Low	Low	Low
6	Galarraga 2020	Small sustainable monetary donation-based incentives to promote physical activity: A randomized controlled trial	Low	High	Low	High	Low
7	Harkins 2017	A trial of financial and social incentives to increase older adults' walking	Low	Low	Low	Low	Low

© 2023 Byrne M et al. *JAMA Network Open*.

8	Haydon 2022	Kindness interventions for early-stage breast cancer survivors: An online, pilot randomized controlled trial	Unclear	Low	Low	Low	Low
9	Hu 2022	Peer charity donation, gratitude, and self-esteem among left-behind children	Low	High	Unclear	Low	Low
10	Lepore 2014	Comparing standard versus prosocial internet support groups for patients with breast cancer: a randomized controlled trial of the helper therapy principle	Low	Low	Low	Low	Low
11	LuengoKanacri 2020	Cross-national evidences of a school-based universal programme for promoting prosocial behaviours in peer interactions: Main theoretical communalities and local unicity	High	High	Low	Low	Low
12	Miles 2022	Using prosocial behavior to safeguard mental health and foster emotional well-being during the COVID-19 pandemic: A registered report of a randomized trial	Low	Low	High	Low	Low
13	Ouweneel 2014	On being grateful and kind: results of two randomized controlled trials on study-related emotions and academic engagement	Unclear	High	Low	Low	Low

© 2023 Byrne M et al. *JAMA Network Open*.

14	Regan 2022	The genomic impact of kindness to self vs. others: A randomized controlled trial	Low	Low	Low	High	Low
15	Rini 2014	Harnessing benefits of helping others: a randomized controlled trial testing expressive helping to address survivorship problems after hematopoietic stem cell transplant	Low	Low	Low	Low	Low
16	Sarason 1993	Increasing participation of blood donors in a bone-marrow registry	Low	Low	Low	Low	Low
17	Schreier 2013	Effect of volunteering on risk factors for cardiovascular disease in adolescents: a randomized controlled trial	Low	High	Low	HIgh	Low
18	Shillington 2021 I	Kindness as an Intervention for Student Social Interaction Anxiety, Affect, and Mood: The KISS of Kindness Study	Low	Low	Low	High	Low
19	Shillington 2021 II	Kindness as an Intervention for Student Social Interaction Anxiety, Resilience, Affect, and Mood: The KISS of Kindness Study II	Low	High	Low	Low	Low
20	Sumida 2014	The 'donations for decreased ALT (D4D)' prosocial behavior incentive scheme for NAFLD patients	Unclear	Low	Low	Low	Low

© 2023 Byrne M et al. *JAMA Network Open*.

21	Sung 2022	Development of a Psychometric Tool to Measure Community Solidarity Among Sexual Minorities: Evidence From a Pay-it-Forward Randomized Controlled Trial	Low	Low	Low	Low	Low
22	Tang 2023	A Pay-It-Forward Approach to Improve Chlamydia and Gonorrhea Testing Uptake Among Female Sex Workers in China: Venue-Based Superiority Cluster Randomized Controlled Trial	High	Low	Low	Low	Low
23	Varma 2022	Prosocial behavior promotes positive emotion during the COVID-19 pandemic	Unclear	Low	Low	Low	Low
24	Wieners 2021	To whom should i be kind? A randomized trial about kindness for strong and weak social ties on mental wellbeing and its specific mechanisms of change	Low	Low	High	High	Low
25	Williamson 2017	Helping Yourself by Offering Help: Mediators of Expressive Helping in Survivors of Hematopoietic Stem Cell Transplant	Low	Low	Low	Low	Low
26	Yang 2020	Pay-it-forward gonorrhoea and chlamydia testing among men who have sex with men in China: a randomised controlled trial	Low	High	Low	Low	Low

© 2023 Byrne M et al. *JAMA Network Open*.

eTable 3: ROBINS-I Tool Results for Non-Randomized Studies

	Lead Author / Year	Title	Overall Bias	Bias due to confounding	Bias in selection of participants	Bias in classification of interventions	Bias due to deviations from intended interventions	Bias due to missing data	Bias in measurement of outcomes	Bias in selection of reported result
1	Li 2019	Pay-it-forward strategy to enhance uptake of dual gonorrhea and chlamydia testing among men who have sex with men in China: a pragmatic, quasi-experimental study	Low	Low	Moderate	Low	Low	Low	Low	Low
2	Ngai 2021	Effectiveness of a school-based programme of animal-assisted humane education in Hong Kong for the promotion of social and emotional learning: A quasi-experimental pilot study	Moderate	Moderate	High	Low	Low	Low	Moderate	Low
3	Vliek 2014	I want to behave prosocially and I can choose to do so: Effectiveness of TIGER (Kanjertraining) in 8- to 11-year-olds	Low	Low	Moderate	Low	Low	Low	Low	Low
4	Wu 2022	Effectiveness of a pay-it-forward intervention compared with user-paid vaccination to improve influenza vaccine uptake and community engagement among children and older adults in China: a quasi-experimental pragmatic trial	Low	Low	Moderate	Low	Low	Low	Low	Low

© 2023 Byrne M et al. *JAMA Network Open*.

eTable 4. GRADE Assessment and Certainty of Evidence for Random Effects Meta-Analysis

Certainty of Evidence							Certainty of Evidence	Importance
**Number of studies**	**Study Design**	**Risk of bias**	**Consistency**	**Directness**	**Precision**	**Other Considerations**		
**Studies assessing the impact of paying-it-forward on improving health outcomes (Li 2019[32]; Tang 2023[36]; Wu 2022[27]; Yang 2020[37])**								
4	Randomized control trials	Mild	No inconsistency	Some indirectness	Mild imprecision	Health outcomes were varied (e.g., vaccine uptake and sexually transmitted disease test uptake)	Moderate	Important

© 2023 Byrne M et al. *JAMA Network Open*.

eTable 5: Considerations for enhancing the effect of prosocial interventions

**Level**	**Considerations**
Individual level Factors	• The motivation and benefit to the giver should be carefully considered. Higher levels of intrinsic motivation can increase the positive effects of the intervention and ideally provide enough benefit to the giver that he or she will continue to engage in this prosocial behavior.<br>• An individuals' available resources, health and psychological wellbeing can also impact one's ability to benefit from a prosocial intervention. Future interventions should consider the baseline of such factors among participants
Interpersonal and contextual factors	• According to the findings of this review, tying in a connection to the recipient and community support can effectively improve the likelihood improving a desired health or medical outcome using a prosocial intervention.
Contextual factors	• When implementing a prosocial intervention, it is essential the researchers consider the overall atmosphere that they are creating for the participants in both the treatment and control group.<br>• An atmosphere that is overly research focused, positive, or negative could impact results, and affect successful implementation of this intervention in other settings.
Nature of prosocial interventions	• Certain prosocial interventions had greater effectiveness than others; including those that included expressions of kindness, charitable donations, paying-it-forward (moderate certainty of evidence), as well as acts of kindness, prosocial spending, blood donation and volunteering time (low certainty of evidence).<br>• These might be due to individual, interpersonal, and contextual factors highlighted above, which should be considered in future implementation.

© 2023 Byrne M et al. *JAMA Network Open*.

eFigure. Meta-analysis of strong kindness versus non-kindness for the weighted mean difference (WMD) for the effect on a) depressive symptoms; b) anxiety symptoms; c) of positive; d) of negative; e) psychological wellbeing.

a) Depressive symptoms

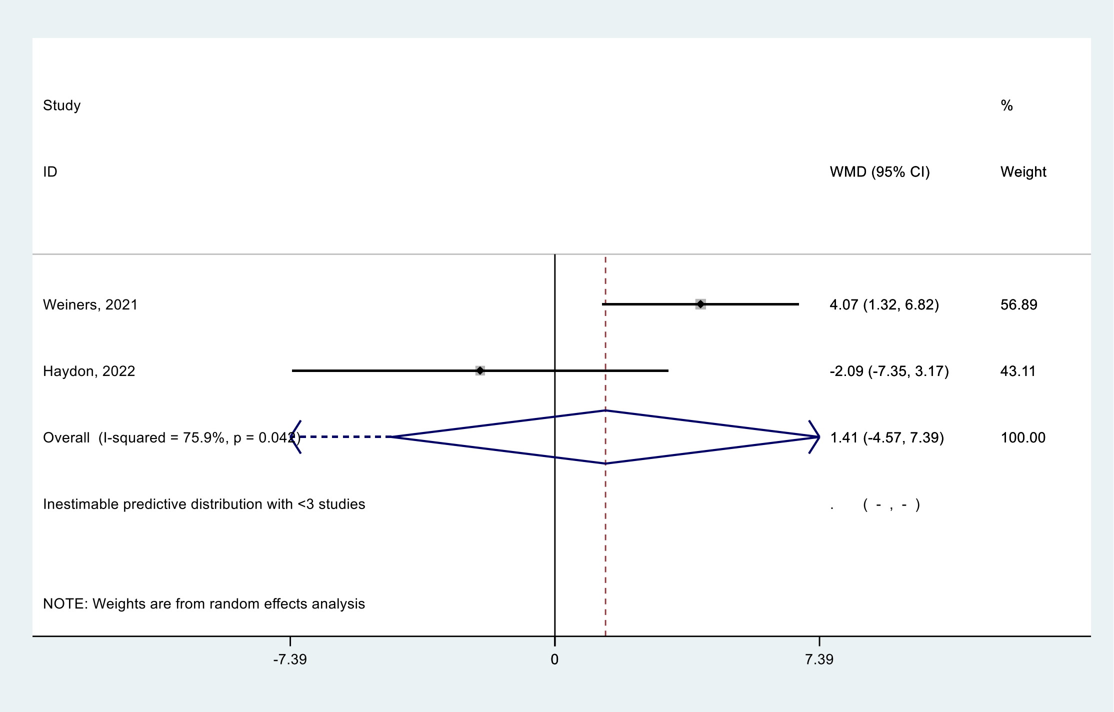

b) Anxiety symptoms

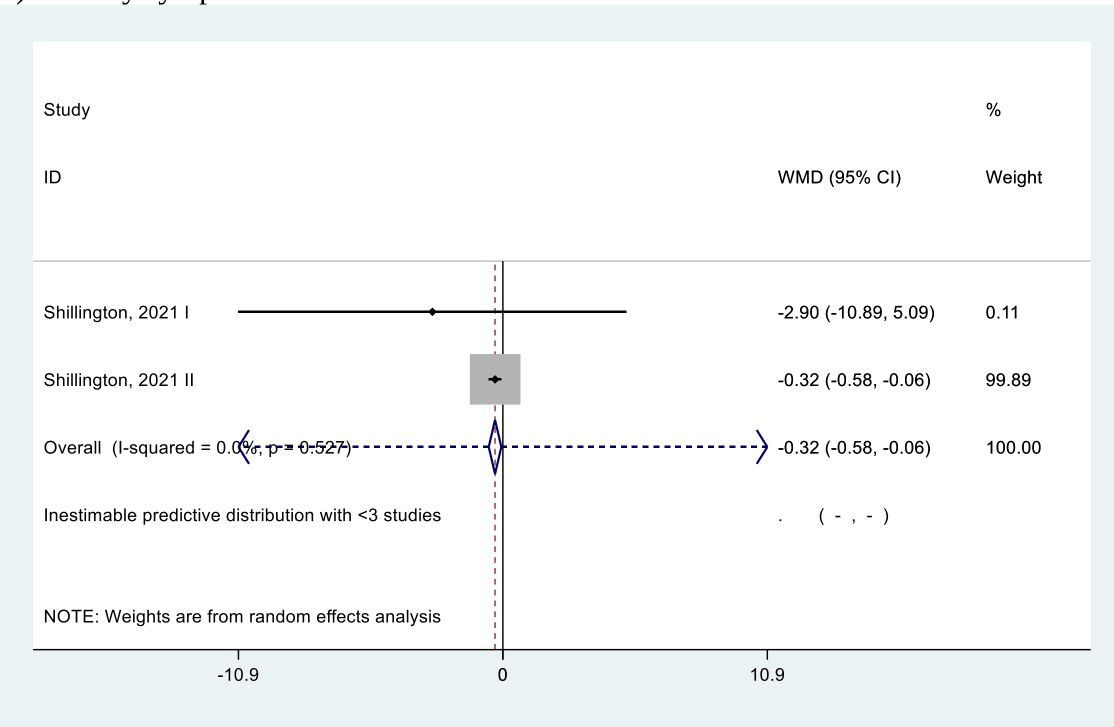

© 2023 Byrne M et al. *JAMA Network Open*.

c) Positive affect

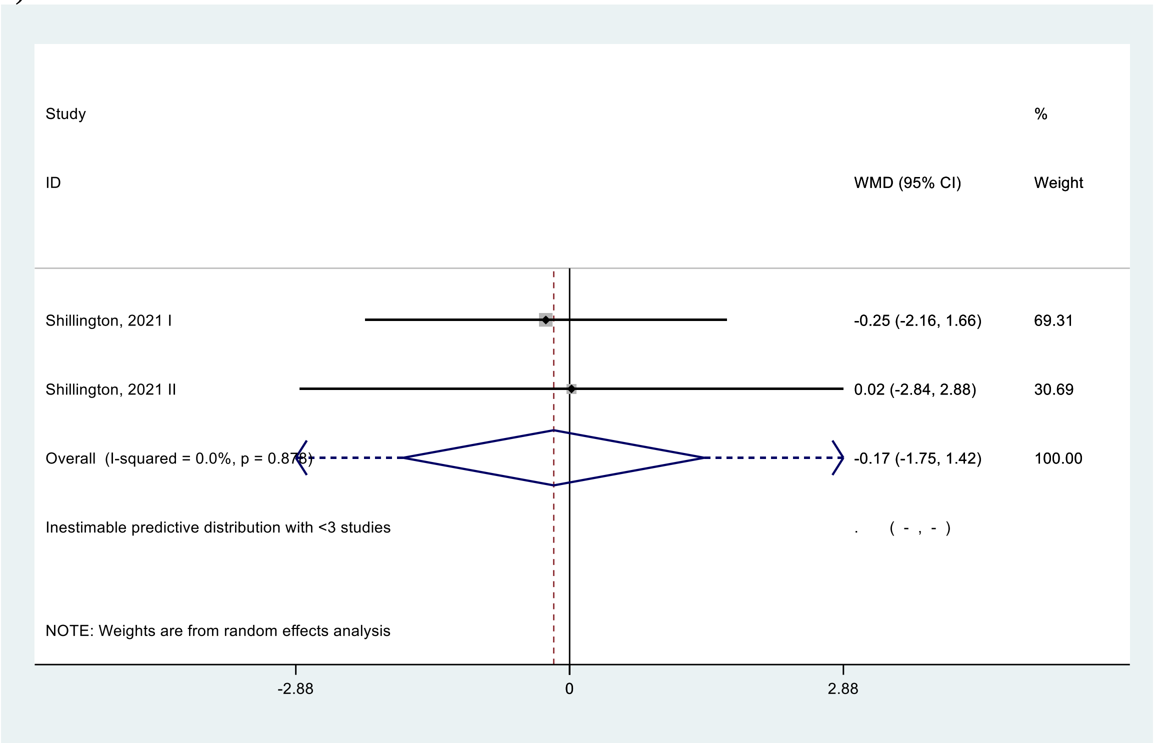

d) Negative effect

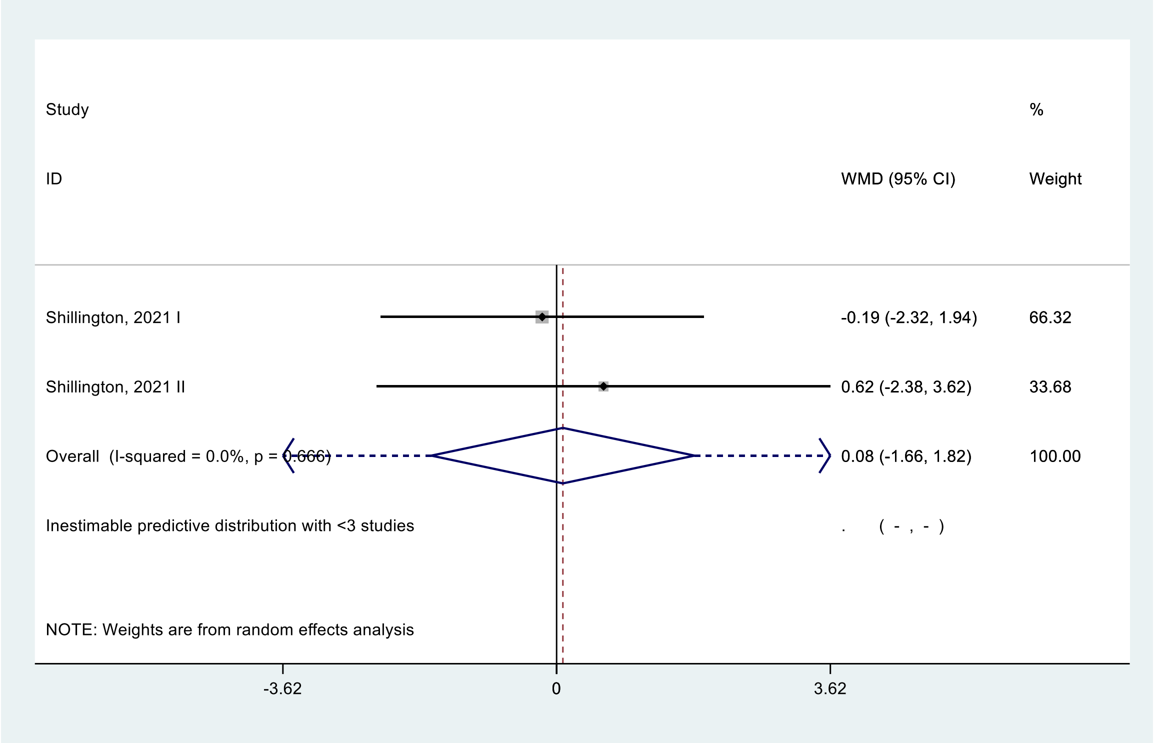

© 2023 Byrne M et al. *JAMA Network Open*.

e) Psychological well-being

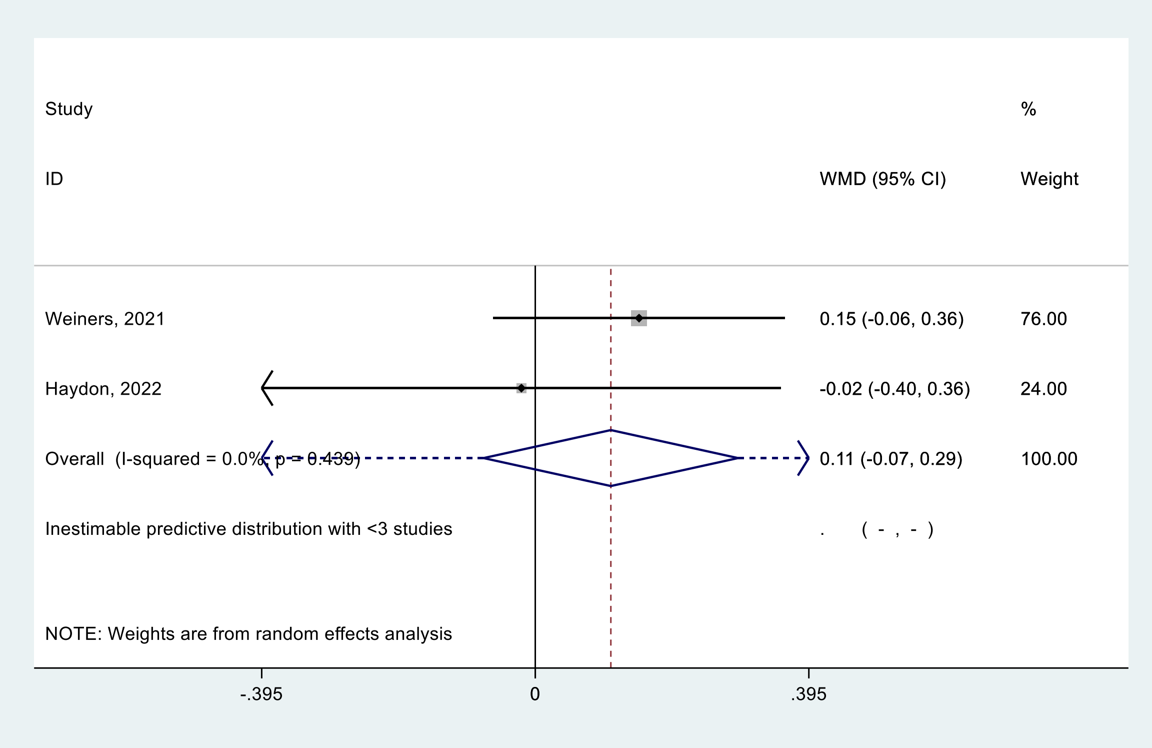

© 2023 Byrne M et al. *JAMA Network Open*.
