# Supplementary material for: Prosocial Interventions and Health Outcomes: A Systematic Review and Meta-Analysis
Source: JAMA Netw Open. 2023 Dec 8;6(12):e2346789. doi: 10.1001/jamanetworkopen.2023.46789 (PMC10709779; doi:10.1001/jamanetworkopen.2023.46789)
Supplement: Supplement 2. — Data Sharing Statement [file jamanetwopen-e2346789-s002.pdf]

Data Sharing Statement

Byrne. Prosocial Interventions and Health Outcomes. *JAMA Netw Open*. Published December 08, 2023. doi:10.1001/jamanetworkopen.2023.46789

Data
Data available: No

Additional Information
Explanation for why data not available: The data in this meta-analysis is secondary data and we do not have any patient data to share.
